# Using social network methods to reach out-of-care or ART-nonadherent HIV+ injection drug users in Russia: addressing a gap in the treatment cascade

**DOI:** 10.7448/IAS.17.4.19594

**Published:** 2014-11-02

**Authors:** Yuri Amirkhanian, Jeffrey Kelly, Anna Kuznetsova, Anastasia Meylakhs, Alexey Yakovlev, Vladimir Musatov, Nikolay Chaika

**Affiliations:** 1 Medical College of Wisconsin, Center for AIDS Intervention Research (CAIR), Milwaukee, WI, USA; 2 Botkin Hospital for Infectious Diseases, Interdisciplinary Center for AIDS Research (ICART), St. Petersburg, Russian Federation; 3 Information, St. Petersburg Pasteur Institute, St. Petersburg, Russian Federation

## Abstract

**Introduction:**

HIV treatment to reduce downstream HIV incidence and to decrease disease mortality and morbidity at a population level both require that hidden, out-of-care people living with HIV (PLH) in the community be reached and engaged to enter care. This research evaluated the feasibility of reaching out-of-care or non-adherent PLH through members of their social networks in St Petersburg, Russia.

**Materials and Methods:**

To recruit a social network sample of HIV-positive injection drug users, 16 HIV+ seeds were enrolled into the study through PLH-oriented websites and online forums using recruitment ads or approached in needle exchange sites. Interested persons called the study phone number and completed a brief eligibility interview. Seed inclusion criteria were HIV+ status, being 18 years or older, having ever injected drugs, and having not visited an HIV doctor in the past 6 months. Seeds provided blood specimens tested for HIV to confirm their self-reported status. Eligible seeds were enrolled, completed brief network elicitation interview, and were asked to invite their own HIV+ friends into the study. Incentives were provided as compensation for participants’ time and additional smaller incentives were provided for inviting each HIV+ network member to also participate. The seed's PLH friends established the first ring of participants who, in turn were asked to invite their own PLH friends (second ring). All study participants completed assessment of psychosocial wellbeing and sexual and injection-related HIV risk behaviour. Blood samples were collected from all participants to confirm their HIV+ status.

**Results:**

Through this chain referral process, the initial 16 seeds led to the enrolment of a total of 66 PLH from the community (mean=4 per initial seed), most of whom – like the seed – were not presently in HIV care or were ART non-adherent.

**Conclusions:**

Implementation of treatment cascade goals requires complementing conventional paths of identifying PLH with feasible and effective community-based approaches such as described in this study. This research establishes that PLH are connected in their day-to-day social networks with other HIV+ persons and shows that social network methods can be employed to reach infected persons through their connections with other PLH. This method has the potential to expand the reach of medical care efforts and ART uptake.

